# Navigating Neurological and Cardiac Complexities: A Case Study on Viral Meningoencephalitis in a Patient With Ischemic Heart Disease

**DOI:** 10.7759/cureus.52763

**Published:** 2024-01-22

**Authors:** Han Grezenko, Faryal Zafar, Eemaz Nathaniel, Guillermo Aguirre, Shariq K Baluch, Muhammad Abubakar

**Affiliations:** 1 Translational Neuroscience, Barrow Neurological Institute, Phoenix, USA; 2 Medicine, Ziauddin University, Karachi, PAK; 3 Clinical Research, Harvard Medical School, Boston, USA; 4 Internal Medicine, Ignacio A. Santos School of Medicine, Ciudad de México, MEX; 5 Internal Medicine, Universidad Autonoma de Guadalajara, Guadalajara, MEX; 6 Internal Medicine, Wah Medical College, Wah, PAK

**Keywords:** personalized medicine, cardiology, neurology, case report, ischemic heart disease, viral meningoencephalitis

## Abstract

We present a case of viral meningoencephalitis in a 40-year-old male with ischemic heart disease, a combination that is rare and presents unique diagnostic and therapeutic challenges. The patient's symptoms included high-grade fever, severe headache, projectile vomiting, and altered consciousness. The diagnosis was supported by MRI and CSF analysis. Management, complicated by the patient's cardiac condition, required a personalized approach, including antiviral therapy, corticosteroids, and vigilant monitoring of cardiac and neurological status. Treatment adjustments were made in response to the patient's evolving condition, leading to improvement within a week. This case underscores the need for a multidisciplinary approach in such complex scenarios, highlighting the significance of tailored care for patients with neurological symptoms and concurrent cardiac comorbidities. The report contributes to the literature on managing meningoencephalitis in patients with significant cardiac histories, underscoring personalized medicine's role in successful outcomes.

## Introduction

Meningoencephalitis, characterized by brain inflammation and meninges inflammation, presents a significant clinical challenge due to its varied etiologies and presentations [1]. While relatively rare in adults, its occurrence carries a considerable risk of morbidity and mortality, necessitating prompt diagnosis and treatment. The annual incidence of meningoencephalitis in adults is estimated at 0.5-1 case per 100,000 in the United States and 0.2-0.4 cases per 100,000 in Europe. In Africa, particularly in the "meningitis belt," the prevalence is notably higher, with annual incidence rates ranging from seven to 20 cases per 100,000 adults. The Asia Pacific region reports an incidence of approximately 0.3 cases per 100,000 adults [2]. Meningoencephalitis commonly affects the central nervous system, but its manifestation can involve multiple systems, adding to the diagnostic complexity [3].

This case report details an unusual presentation of viral meningoencephalitis in a 40-year-old male who is a known case of ischemic heart disease (IHD). The intersection of viral meningoencephalitis with a significant cardiac history is rare and presents unique challenges in diagnosis and management. This case report aims to analyze this patient's diagnosis, treatment, and outcome. This report aims to enhance understanding and guide clinical decision-making in similar complex cases, contributing to the broader medical knowledge base in neurology and cardiology.

## Case presentation

A 40-year-old male with a known history of IHD and a past smoker presented with a two-week history of intermittent high-grade fever and a sudden episode of altered level of consciousness (ALOC). His past medical history was significant for a prosthetic valve implantation four years prior with a good prognosis. He had a 10-year history of smoking, one pack per day, which he quit five years ago. The patient's fever was associated with chills, generalized body aches, severe headache, and frequent episodes of projectile vomiting containing food particles.

On examination, vital signs revealed a blood pressure of 110/90 mmHg, respiratory rate of 22 breaths per minute, pulse of 96 beats per minute, and fever of 102°F. Neurological examination showed a Glasgow Coma Scale score of 4/15 (E1M2V1); the right plantar response was static, and the left was going down. Meningeal irritation signs were mildly positive, with mild neck stiffness and positive Brudzinski's sign and Kernig's sign. This clinical picture raised the suspicion of meningitis.

**Figure 1:**
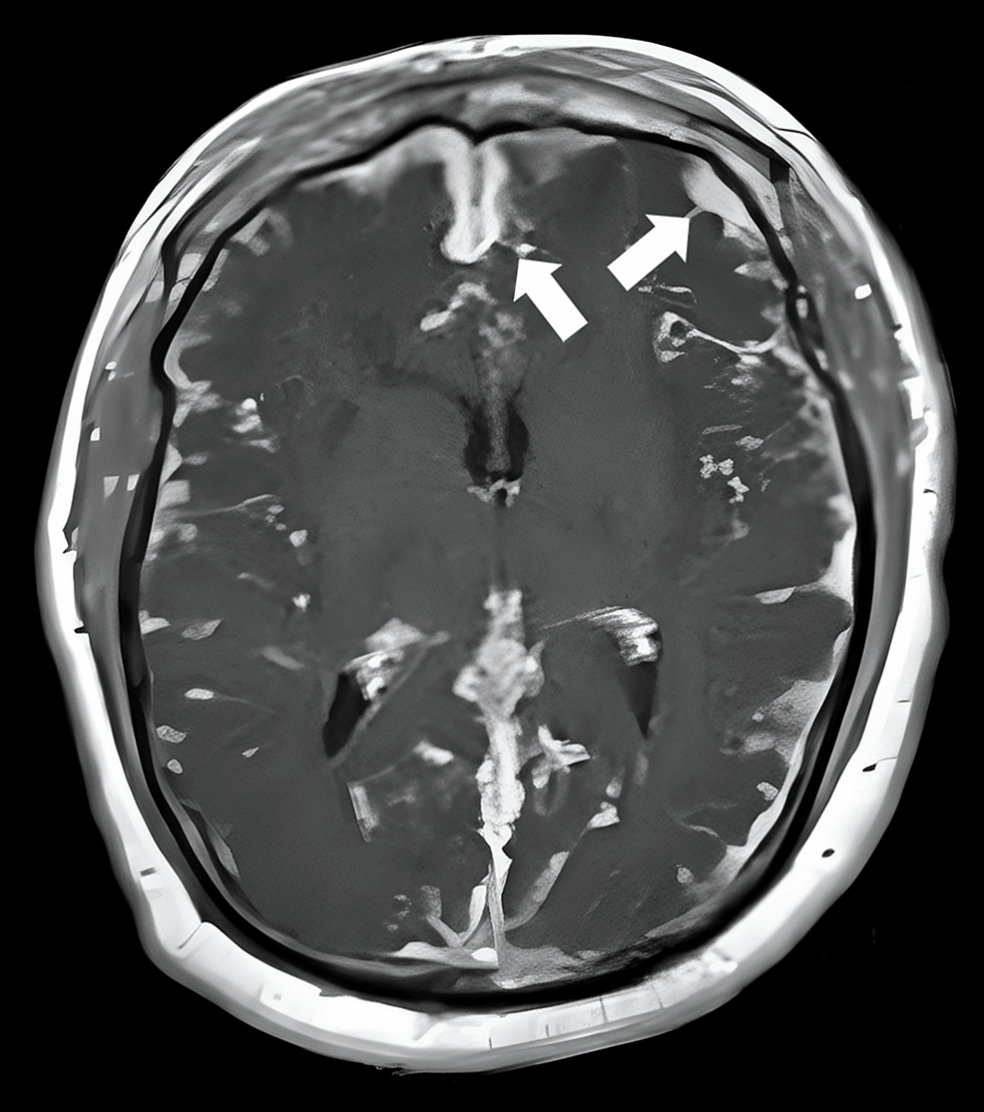
The MRI image elaborating leptomeningeal enhancement, as marked by the arrows.

Baselines and magnetic resonance imaging (MRI) were conducted to confirm the diagnosis. The MRI findings included leptomeningeal enhancement, increased T2/fluid-attenuated inversion recovery (FLAIR) signal in specific brain regions, microhemorrhages, diffusion abnormalities, and contrast enhancement of the choroid plexus. Infarcts from pre-existing IHD were also noted. These findings were suggestive of meningoencephalitis. Findings are elaborated in Figure 1.

A comprehensive laboratory test set was performed, including a complete blood count, coagulation profile, renal and liver function tests, serum electrolytes, and inflammatory markers. These results helped assess the patient's overall health status and rule out other potential causes of his symptoms. A complete blood workup is given in Table 1.

**Table 1:** Complete blood workup of the patient. INR: international normalized ratio; APTT: activated partial thromboplastin time; WBC: white blood cells; RBC: red blood cells; HCT: hematocrit; MCV: mean corpuscular volume; MCH: mean corpuscular hemoglobin; MCHC: mean corpuscular hemoglobin concentration; ALT: alanine transaminase; AST: aspartate aminotransferase; ALP: alkaline phosphatase; ESR: erythrocyte sedimentation rate.

Coagulation profile		
Tests	Values	Reference range
Prothrombin time-control	12	10-14 seconds
Prothrombin time-patient	15	Up to 13 seconds
INR	1.25	0.9-1.3
Control time	32	25-35 seconds
APTT	34	Up to 31 seconds
Hemogram		
WBC count	13.9	4-11 x10^9^/L
Total RBC	4.6	3.8-5.2 x10^12^/l
Hemoglobin	13.5	13-18 (g/dL)
HCT	39.3	35-46%
MCV	85.4	77-95 fl
MCH	29.3	26-32 (pg)
MCHC	34.4	32-36 (g/dL)
Platelets	478	150-400 x10^9^/L
Neutrophils	85.6	40-80%
Lymphocytes	7.9	20-40%
Renal function tests		
Urea	34	10-50 mg/dl
Serum creatinine	0.7	0.5-0.9 mg/dl
Liver function tests		
Bilirubin total	0.38	0.3-1.2 mg/dl
ALT	40	Up to 40 U/L
AST	46	Up to 40 U/L
ALP	109	40-120 U/L
Serum electrolytes		
Sodium	132	135-145 mmol/L
Potassium	3.3	3.5-5 mmol/L
Chloride	94	98-107 mmol/L
Inflammatory markers		
ESR	6	0-25 mm/1^st^ hour

A lumbar puncture for the cerebrospinal fluid (CSF) examination revealed elevated protein and normal glucose levels, with lymphocytic predominance. This supported the diagnosis of viral meningoencephalitis. Findings of lumber puncture are given in Table 2.

**Table 2:** Results of cerebrospinal fluid examination.

Physical examination		
Specific contents	Values	Range
Color	Colorless	Colorless
Coagulum	Not seen	Nil
Turbidity	Negative	Negative
Chemical examination		
Glucose	93	50-80 mg/dL
Protein	177	<45 mg/dL
Microscopic examination		
Red blood cells	Nil	Nil
White blood cells	3	0-10/cubic millimeter
Neutrophils	Nil	Nil
Lymphocytes	100	70%

The differential diagnosis included bacterial meningitis, autoimmune encephalitis, and other viral infections. The clinical presentation, CSF analysis, and MRI findings were instrumental in differentiating these conditions. The patient was managed with a combination of antiviral therapy (injection acyclovir 500 mg thrice daily), antibiotics (injection ceftriaxone 2 g IV twice daily) due to the raised neutrophil count suggesting a superimposed bacterial infection, corticosteroid therapy (injection dexamethasone IV twice daily) for cerebral edema, and symptomatic support, including injection omeprazole 40 mg IV once daily and chest therapy. The rationale for this regimen was based on the presumed viral etiology, with acyclovir targeting herpes simplex virus and the need for broad coverage against possible bacterial superinfection.

The patient's condition fluctuated during the initial three days of treatment, necessitating close monitoring and adjustments in the management plan. Vitals were monitored every two hours, and oxygen support was provided. Challenges included balancing adequate hydration and avoiding volume overload due to his cardiac history. After one week of treatment, there was a noted improvement in the patient's clinical status but continued monitoring and supportive care were planned.

## Discussion

Viral meningoencephalitis typically presents with fever, headache, altered mental status, and signs of meningeal irritation, as seen in our patient. However, what sets this case apart is the occurrence of these symptoms in a relatively young patient with a significant history of IHD and a prosthetic heart valve. While cases of viral meningoencephalitis are documented, its presentation in patients with such cardiac histories is rare. Literature review reveals that while meningoencephalitis is more commonly reported in older adults, especially those over 60 [1], its occurrence in younger adults with IHD is not well-documented, making this case particularly noteworthy.

The management of viral meningoencephalitis in patients with pre-existing conditions like IHD poses unique challenges [2]. In managing the patient's fluid balance, a precise and cardiac-focused approach was paramount. Continuous hemodynamic monitoring was integral, involving regular echocardiographic assessments and central venous pressure measurements to meticulously gauge and maintain optimal fluid homeostasis. This strategy was pivotal in circumventing the risks of both dehydration and cardiac volume overload, critical in a patient with underlying IHD. Fluid management was dynamically adjusted, responsive to the patient’s real-time clinical status and hemodynamic indicators.

Concurrently, corticosteroid therapy necessitated a comprehensive and interdisciplinary risk-benefit evaluation, engaging both cardiology and neurology specialists. Recognizing the concurrent risks of cerebral edema and the potential for exacerbating heart failure, the corticosteroid regimen was judiciously determined. Initiation at a conservative dosage, with vigilant monitoring for any indications of cardiac compromise, was a critical component of the treatment strategy. This proactive and attentive approach underscored the imperative of individualized pharmacotherapy and the necessity for flexible, responsive decision-making in clinical scenarios characterized by overlapping neurological and cardiac complexities.

Diagnosing viral meningoencephalitis in a patient with IHD requires a multifaceted approach, utilizing MRI and CSF analysis to distinguish it from conditions like bacterial meningitis or autoimmune encephalitis [4,5]. Empirical treatment with acyclovir, targeting herpes simplex virus, was coupled with broad-spectrum antibiotics to address potential bacterial superinfection, considering the patient's IHD and prosthetic valves [6]. This case highlighted the necessity for a multidisciplinary care team, including neurologists, cardiologists, and intensivists, to manage the patient's fluctuating condition and the intricate neurological-cardiac interplay. It emphasizes the rarity of such presentations, stressing the need for high clinical suspicion of viral meningoencephalitis in patients with neurological symptoms, regardless of age or comorbidities, and adds to the literature on managing meningoencephalitis in patients with complex cardiac histories [7].

## Conclusions

In conclusion, this case report of viral meningoencephalitis in a 40-year-old male with IHD underscores the complexities of managing neurological conditions in patients with significant cardiac histories. The integration of antiviral therapy with careful cardiac management exemplifies the necessity for personalized, multidisciplinary approaches in such atypical presentations. This case highlights the importance of adaptability and patient-specific treatment strategies in achieving favorable outcomes, contributing valuable insights into the interplay between neurological infections and cardiac comorbidities. It reinforces the essential role of personalized medicine in complex clinical scenarios, guiding future clinical decision-making in similar cases.

## References

[1] (2023). Cleveland Clinic. Meningoencephalitis. https://my.clevelandclinic.org/health/diseases/25157-meningoencephalitis.

[2] Solomon T, Hart IJ, Beeching NJ (2007). Viral encephalitis: a clinician's guide. Pract Neurol.

[3] (2023). Verywell Health. What is meningoencephalitis?. https://www.verywellhealth.com/meningoencephalitis-5082300.

[4] Mailles A, Stahl JP (2009). Infectious encephalitis in France in 2007: a national prospective study. Clin Infect Dis.

[5] Venkatesan A, Geocadin RG (2014). Diagnosis and management of acute encephalitis: a practical approach. Neurol Clin Pract.

[6] Cooper J, Kierans C, Defres S, Easton A, Kneen R, Solomon T (2017). Care beyond the hospital ward: understanding the socio-medical trajectory of herpes simplex virus encephalitis. BMC Health Serv Res.

[7] Lee KS, Park DI, Lee J, Oh O, Kim N, Nam G (2023). Relationship between comorbidity and health outcomes in patients with heart failure: a systematic review and meta-analysis. BMC Cardiovasc Disord.

